# Rectal Colonization and Nosocomial Transmission of Carbapenem-Resistant *Acinetobacter baumannii* in an Intensive Care Unit, Southwest Nigeria

**DOI:** 10.3389/fmed.2022.846051

**Published:** 2022-03-07

**Authors:** Erkison Ewomazino Odih, Emmanuel Oladayo Irek, Temitope O. Obadare, Anderson O. Oaikhena, Ayorinde O. Afolayan, Anthony Underwood, Anthony T. Adenekan, Veronica O. Ogunleye, Silvia Argimon, Anders Dalsgaard, David M. Aanensen, Iruka N. Okeke, A. Oladipo Aboderin

**Affiliations:** ^1^Department of Pharmaceutical Microbiology, Faculty of Pharmacy, Global Health Research Unit for the Genomic Surveillance of Antimicrobial Resistance, University of Ibadan, Oyo, Nigeria; ^2^Department of Veterinary and Animal Sciences, Faculty of Health and Medical Sciences, University of Copenhagen, Copenhagen, Denmark; ^3^Department of Medical Microbiology and Parasitology, Obafemi Awolowo University Teaching Hospitals Complex, Ife, Nigeria; ^4^Centre for Genomic Pathogen Surveillance, Wellcome Sanger Institute, Cambridge, United Kingdom; ^5^Big Data Institute, University of Oxford, Oxford, United Kingdom; ^6^Department of Anaesthesia and Intensive Care, Obafemi Awolowo University, Ife, Nigeria; ^7^University College Hospital, Ibadan, Nigeria; ^8^Department of Medical Microbiology and Parasitology, Obafemi Awolowo University, Ife, Nigeria

**Keywords:** *Acinetobacter baumannii*, rectal colonization, nosocomial, hospital-acquired infections, carbapenem-resistant *Acinetobacter baumannii*, antimicrobial resistance

## Abstract

**Background:**

*Acinetobacter baumannii* are of major human health importance because they cause life-threatening nosocomial infections and often are highly resistant to antimicrobials. Specific multidrug-resistant *A. baumannii* lineages are implicated in hospital outbreaks globally. We retrospectively investigated a suspected outbreak of carbapenem-resistant *A. baumannii* (CRAB) colonizing patients in an intensive care unit (ICU) of a tertiary hospital in Southwest Nigeria where genomic surveillance of *Acinetobacter* has hitherto not been conducted.

**Methods:**

A prospective observational study was conducted among all patients admitted to the ICU between August 2017 and June 2018. *Acinetobacter* species were isolated from rectal swabs and verified phenotypically with the Biomerieux Vitek 2 system. Whole genome sequencing (WGS) was performed on the Illumina platform to characterize isolates from a suspected outbreak during the study period. Phylogenetic analysis, multilocus sequence typing, and antimicrobial resistance gene prediction were carried out *in silico*.

**Results:**

*Acinetobacter* isolates belonging to the *A. baumannii* complex were recovered from 20 (18.5%) ICU patients. Single nucleotide polymorphism (SNP) analysis and epidemiological information revealed a putative outbreak clone comprising seven CRAB strains belonging to the globally disseminated international clone (IC) 2. These isolates had ≤2 SNP differences, identical antimicrobial resistance and virulence genes, and were all ST1114/1841.

**Conclusion:**

We report a carbapenem-resistant IC2 *A. baumannii* clone causing an outbreak in an ICU in Nigeria. The study findings underscore the need to strengthen the capacity to detect *A. baumannii* in human clinical samples in Nigeria and assess which interventions can effectively mitigate CRAB transmission in Nigerian hospital settings.

## Introduction

*Acinetobacter baumannii* are opportunistic pathogens of increasing global public health concern. These Gram-negative organisms are widely implicated in life-threatening drug-resistant infections in hospitalized patients. *A. baumannii* are often introduced in patients through contaminated medical devices, and infections include pneumonia, urinary tract infection, bloodstream infection, endocarditis, meningitis and wound infection (1). More so, these infections are often prone to epidemic spread within hospital settings, facilitated by the excellent ability of *A. baumannii* to survive in harsh hospital environments (2).

High resistance to desiccation, efficient biofilm-forming ability and the frequent carriage of resistance determinants to both antimicrobials and commonly used disinfectants mean that *A. baumannii* are especially well suited for survival in the hospital environment, including hospital surfaces, utensils and equipment, invasive medical devices, as well as hospital personnel (3–6). Prolonged survival allows *A. baumannii* to excel as nosocomial pathogens and makes their transmission difficult to control within hospitals. Healthcare personnel play important roles in this cross-transmission within the hospital as they get frequently contaminated *via* contact with infected or colonized patients as well as contaminated abiotic surfaces (4). Nosocomial *A. baumannii* infections are associated with high mortality, lengthened hospital stay and increased hospital costs (7).

The clinical burden of *A. baumannii* is further worsened by their frequent carriage of multiple resistance determinants, limiting treatment options and worsening outcomes. Their highly plastic genomes facilitate the acquisition and maintenance of genes conferring resistance to different antimicrobials, including the carbapenems which are last-line antimicrobials for treatment of these infections (1, 8). The limited treatment options available for carbapenem-resistant *A. baumannii* (CRAB) infections caused the World Health Organization to prioritize CRAB as number one on the priority list of pathogens for which new antimicrobials are urgently needed (9). Mechanisms of resistance to carbapenems in *A. baumannii* include overexpression of efflux pumps, membrane porin modification and, most prominently, possession of resistance genes, particularly the OXA- and NDM-type carbapenemases, which are borne on either plasmids or chromosomes (2, 10, 11). Resistance to carbapenems is associated with the successful and globally disseminated major *A. baumannii* clones, international clones (ICs) 1–3, with very high resistance rates reported (12). These clones, with IC2 being the most successful and widely described, are extensively drug-resistant and are the most frequently reported cause of CRAB outbreaks in hospital settings worldwide (2). Nevertheless, high carbapenem resistance rates have been reported in non-IC *A. baumannii* clones endemic in several countries (11, 13, 14).

Little is known about the molecular epidemiology and antimicrobial resistance profiles of *A. baumannii* infections in Nigeria, largely due to limited capacity for the isolation, identification and antimicrobial susceptibility testing of *A. baumannii* in routine clinical laboratories, as well as poor access to molecular characterization techniques. However, a few reports exist describing CRAB causing clinical infections in hospitals in Nigeria. These studies, one conducted almost 10 years ago in 2012 (15), and another in 2018 (16), reported the detection of *blaOXA*-23 and *blaNDM*-1 carbapenem resistance genes in clinical CRAB isolates mostly from southwestern Nigeria hospitals. Surveillance and understanding of the risk factors for transmission of *A. baumannii* infections within hospital intensive care units (ICUs) is critical for establishing effective infection control and prevention measures to curtail further spread between high-risk immunosuppressed patients. Evidence suggests that even the seemingly harmless colonization of body sites, including the axilla, pharynx and gastrointestinal tract, by *A. baumannii* within ICUs can precede subsequent infection (17). We retrospectively investigated a suspected outbreak of *A. baumannii* in the intensive care unit (ICU) of a tertiary hospital in Southwest Nigeria.

## Materials and Methods

### Ethical Considerations

Ethical approval for the study was granted by the Ethics and Research Committee of the Obafemi Awolowo University Teaching Hospitals Complex, Ife, Nigeria with protocol number ERC/2017/06/13. Participants provided written informed consent before voluntarily participating in the study. Patient confidentiality and anonymity were maintained. Only de-identified patient metadata with no traceability to patients was collected and analyzed.

### Sample Collection and Bacterial Identification

A prospective observational study was conducted to determine the colonization and transmission of CRAB among all new patients admitted into the ICU of the Obafemi Awolowo University Teaching Hospitals Complex (OAUTHC), Ife, Osun State, Nigeria between August 2017 and June 2018. A total of 108 patients were recruited. Rectal swabs were collected from each patient within 48 h of ICU admission and, thereafter, weekly until exit using the protocol for active surveillance cultures by the US Centres for Disease Control and Prevention (18). Acquisition rate was defined as the percentage of patients who acquired CRAB that was absent on admission. CRAB was isolated from the rectal swab samples and preliminarily identified using standard microbiological procedures, and then cryopreserved at −80°C. We also verified all *A. baumannii* isolates from human specimens and associated metadata retrospectively submitted to the then-new Nigerian antimicrobial resistance (AMR) surveillance system by OAUTHC, Ife, Osun State and from the University College Hospital (UCH), Ibadan, located in the adjacent Oyo State. The identities of all presumptive *Acinetobacter* species were verified by culture on CHROMagar™ Acinetobacter media with CHROMagar MDR Supplement CR102 (CHROMagar, Paris, France) and identification on a VITEK 2 automated system (bioMérieux, Inc., Marcy-l’Étoile, France) following manufacturer instructions using GN ID (reference number: 21341) cards. Antimicrobial susceptibility testing was also carried out on the Vitek 2 automated system using the AST N281 (reference number: 414531) cards. Antibiotics tested were ticarcillin/clavulanic acid, piperacillin/tazobactam, ceftazidime, cefoperazone/sulbactam, cefepime, doripenem, imipenem, meropenem, gentamicin, ciprofloxacin, levofloxacin, minocycline, tigecycline and trimethoprim/sulfamethoxazole. Minimum inhibitory concentration values were interpreted according to the Clinical Laboratory Standards Institute guidelines (19).

### DNA Extraction, Library Preparation, and Sequencing

Genomic DNA extraction was carried out using the FastDNA Spin Kit for Soil (MP Biomedicals, Irvine, CA, United States) with protocols modified for bacterial genomic DNA extraction. The *Acinetobacter* isolates were grown overnight in Tryptone Soy Broth (Oxoid, Basingstoke, United Kingdom) and centrifuged to obtain the cell pellets at 6,000 revolutions per minute for 5 min. The pellet was then used for extracting the DNA following the manufacturer’s instructions. Quantification of extracted DNA was done using the Qubit™ dsDNA BR Assay Kit (Invitrogen, Waltham, MA, United States). Library preparation was done using the Covaris LC220 for fragmentation, and the NEBNext Ultra II FS DNA library kit for Illumina with 384-unique indexes (New England Biolabs, Ipswich, MA, United States). The double-stranded DNA libraries (avg. 500 bp) were sequenced using the HiSeq X10 with 150 bp paired-end chemistry (Illumina, San Diego, CA, United States).

### Whole Genome Sequence Analysis

All sequence analyses, except where otherwise stated, were carried out as described in the Global Health Research Unit (GHRU) protocol^1^ using publicly available Nextflow pipelines. *De novo* assembly, species identification, and quality control were carried out using the *De novo* assembly pipeline with default parameters. Quality checks included total bases between 3,340,530–4,776,219 megabase pairs (Mbp), contamination levels <5%, N50 scores >25000, and number of contigs <300; assemblies that “failed” any of these checks were excluded from the downstream analyses.

To determine evolutionary relationships among the isolates, single nucleotide polymorphism (SNP) phylogeny analysis was conducted using the SNP phylogeny pipeline with default parameters. Briefly, Bactinspector^2^ was used to select the closest reference to the *A. baumannii* sequences (Genbank accession: GCA_000830055.1). Reads were mapped to the reference, and variants were called, filtered and concatenated into pseudogenomes. Afterward, the pseudogenomes were aligned and a maximum likelihood tree was constructed from the aligned pseudogenomes with the GTR + G model and 1,000 bootstraps. To determine the clonality of isolates within suspected outbreak clades, we re-selected a reference sequence (NZ_CP016298.1) more closely related to the strains of interest, computed pseudogenome alignments as previously described and calculated the pair-wise SNP distances between the strains based on the aligned pseudogenomes using FastaDist^3^.

Multi-locus sequence types (MLST) of the strains were determined *in silico* as described in the GHRU protocol based on the Oxford and Pasteur MLST schemes (20, 21). The goeBURST software^4^ was used to assign the predicted sequence types (STs) to IC groups based on locus similarities to known international clones (22). The acquired antimicrobial resistance determinants harbored by each of the isolates were also predicted *in silico* as described in the aforementioned GHRU protocol using the Ariba software and the National Center for Biotechnology Information’s (NCBI) Bacterial Antimicrobial Resistance Reference Gene Database^5^. Only predicted genes tagged by the ariba software as “yes” or “yes_nonunique” were regarded as present in the genome.

### Statistical Analysis

Patient clinical data was entered into WHONET 5.6^6^ and cleaned on Microsoft Excel^®^ (Microsoft Corporation, Redmond, WA, United States) spreadsheet. Data was then analyzed using Statistical Package for the Social Science (SPSS^®^, IBM Corp., Armonk, NY, United States) version 20. A descriptive statistical analysis was carried out to summarize the demographic data and results were presented as frequency distribution, percentages, mean, and standard deviation. Risk factors for CRAB-colonization/infection were assessed using bivariate analysis with Chi-square and multivariate analysis with logistic regression. *P*-values <0.05 were considered statistically significant.

## Results

### Study Population and Species Distribution

Rectal swab samples were obtained from 108 patients admitted to the ICU at OAUTHC between August 2017 and June 2018. Carbapenem-resistant *A. baumannii* (CRAB) was recovered from 20 (18.5%) patients. The acquisition rate was 8.3% (8/96), while 12 (11.1%) patients were positive for CRAB within 48 h of admission. Patients that acquired CRAB had seven times the odds of subsequent bloodstream infection (OR = 7.41; 95% CI 2.39–22.92). The mortality rate among CRAB-colonized patients was 50% and the odds of death was almost two times higher among the CRAB-colonized patients compared to patients with no CRAB colonization (OR = 1.84, 95%; CI = 0.69–4.90).

### Rectal Colonization Outbreak in Obafemi Awolowo University Teaching Hospitals Complex Intensive Care Unit Ward Identified by Whole Genome Sequencing

A total of 36 bacterial genomes were analyzed; 20 were isolated from the ICU at OAUTHC between 2017 and 2018 and were part of the suspected rectal colonization outbreak, while the remaining 16 were retrospective isolates submitted to Nigeria’s AMR surveillance system (2017 – 2019) by OAUTHC and UCH. The isolates were identified as *A. baumannii* (34), *A. nosocomialis* (one), and *A. pittii* (one) based on their whole-genome sequences.

**FIGURE 1:**
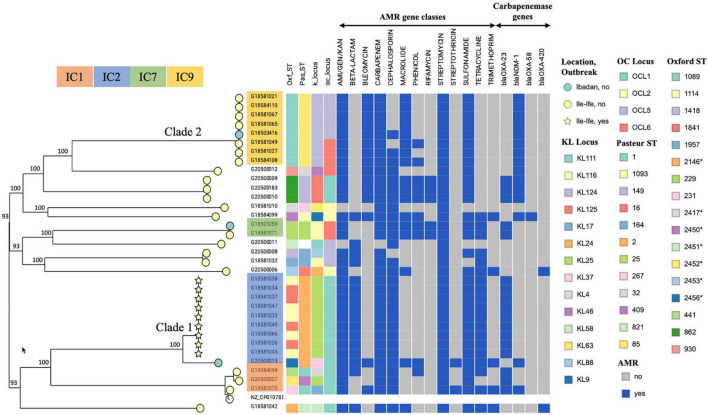
Maximum likelihood phylogeny of *A. baumannii* isolates from OAUTHC, Ife, Osun State and UCH, Ibadan, Oyo State, Nigeria between 2017 and 2019. Sequence types, KL and OC types, classes of antimicrobials to which resistance is conferred by harbored genes, and carbapenem resistance genes are shown. Tree node colors represent isolate collection location; nodes in star shape represent the confirmed outbreak strains. Internal node labels show percentage bootstrap support for all bifurcations. https://microreact.org/project/eWkGUYTxYUJ6Ctv79Lyxj9-acinetobacter-2021-10-03.

The 34 *A. baumannii* strains segregated into clear evolutionarily distinct lineages, with isolates in each lineage belonging to identical STs. Half (17/34; 50.0%) of the isolates belonged to the two major clades observed (clade 1 and clade 2) and three different STs: ST1114 and ST1841 (which we later found to be the same ST but were artifactually different due to a duplicated *gdhB* gene in the strains) in clade 1, and ST1089 in clade 2 (Figure 1).

All nine clade 1 (IC2) strains and five of the eight clade 2 strains were from the OAUTHC ICU. Based on phylogenetic relatedness, timeline of isolation and other epidemiological data, we hypothesize that the clade 1 isolates were part of an outbreak in the ICU, while the others, including those in clade 2, were endemic circulating strains isolated during the same period. After the isolation of the first clade 1 strain on 2nd January 2018, seven more identical strains belonging to this clade were isolated within 3 weeks (between 17th March 2018 and 9th April 2018) of each other from patients in the same ICU. Conversely, and further supporting our hypothesis of repeated introduction, the five clade 2 strains from the ICU were isolated over 42 weeks. The intra-clade SNP distances between the isolates ranged from 0–213 SNPs. Also, one of the clade 2 strains was isolated at another tertiary facility in a different state in southwest Nigeria; the date of isolation was not available for this as well as most of the other non-ICU isolates.

Clade 1 isolates all had an identical AMR and virulence gene composition and resolved into two sub-clades – clade 1A (two isolates) and clade 1B (seven isolates). Each sub-clade had a maximum within-clone distance of two SNPs. The SNP distances between the two sub-clades were ∼48 SNPs. A previous study reported a threshold of ∼2.5 core-genome SNPs for distinguishing outbreak and non-outbreak *A. baumannii* strains (24). We conclude that clade 1B isolates were part of a definite outbreak; however, as there were only 2 clade 1A isolates, it could not be determined if these isolates were also part of an independent and concurrent outbreak in the ICU during the study period.

### Whole Genome Sequencing Resolution Allowed the Identification of Outbreak Strains

As the suspected outbreak isolates within clade 1 were close to identical (≤2 SNPs), we further investigated the reason for the different ST assignments of the isolates within this clade. Based on the Oxford MLST scheme, ST1114 and ST1841 strains are single locus variants with allelic differences in the *gdhB gene*. We retrieved the sequences of these two alleles from the pubMLST database and searched them against the contigs of all clade 1 isolates using BLAST. This revealed two copies of the *gdhB* gene within all nine clade 1 isolates, with each copy sharing 100% identity with either allele 3 or 189. This demonstrated that all clade 1 strains belonged to the same ST and supported our outbreak hypothesis. With repeated runs, however, our MLST pipeline consistently detected allele 189 among ST1841 isolates and allele 3 among the ST1114 isolates.

### Local Epidemiology of *Acinetobacter baumannii* Infections

**FIGURE 2:**
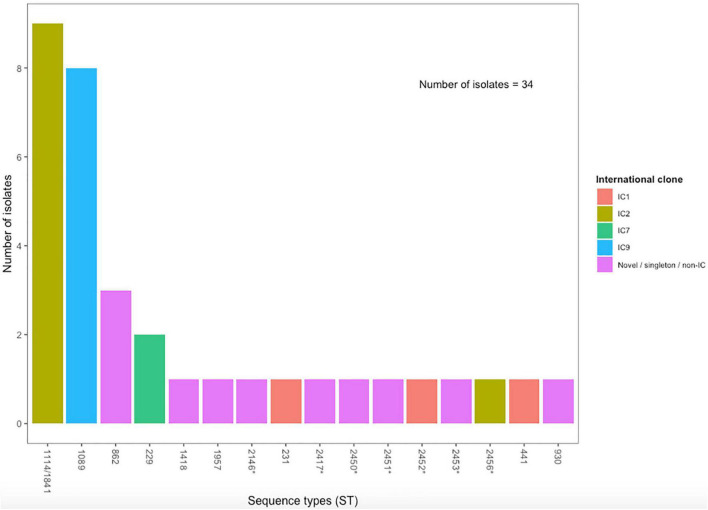
MLST distribution of *A. baumannii* isolates from OAUTHC, Ife, Osun State and UCH, Ibadan, Oyo State, Nigeria between 2017 and 2019. Bars are colored according to the international clone assignment (20, 34). STs with asterisks are novel STs detected in this study.

We characterized the strains based on MLST data to identify the lineages causing *A*. *baumannii* infections in Nigeria. Nine of the 34 (20.6%) *A. baumannii* isolates, and the single *A. pittii* isolate, had novel MLST allelic profiles (Figure 2). Upon submission of these isolates and profiles to the PubMLST database, we found that two of the STs had also been detected in other studies – one in China (ST2417) and the other in Ghana (ST2146) – around the same period and submitted to the database. The other eight novel ST profiles were submitted to the PubMLST database with submission ID BIGSdb_20210908105012_023242_52326 and have been assigned STs (Supplementary Table 1). The nine outbreak *A. baumannii* isolates (ST1114 and ST1841), as well as one of the novel ST strains (G20500013; ST2456), belonged to IC2, while three of the strains belonged to IC1 (ST231, ST441, and the novel ST2451 strain). Other international clones of *A. baumannii* were also present among our strains. However, the majority (19/34; 55.9%) of the *A. baumannii* isolates were singletons, non-major international clones or novel. The eight ST1089 strains clustered with other IC9 sequence types in the PubMLST database, while the two ST229 strains clustered with other IC7 sequence types. Both ST229 isolates were phylogenetically identical and had identical AMR and virulence genes but were isolated from different locations, one from UCH and the other from OAUTHC.

### Antimicrobial Resistance Determinants

**FIGURE 3:**
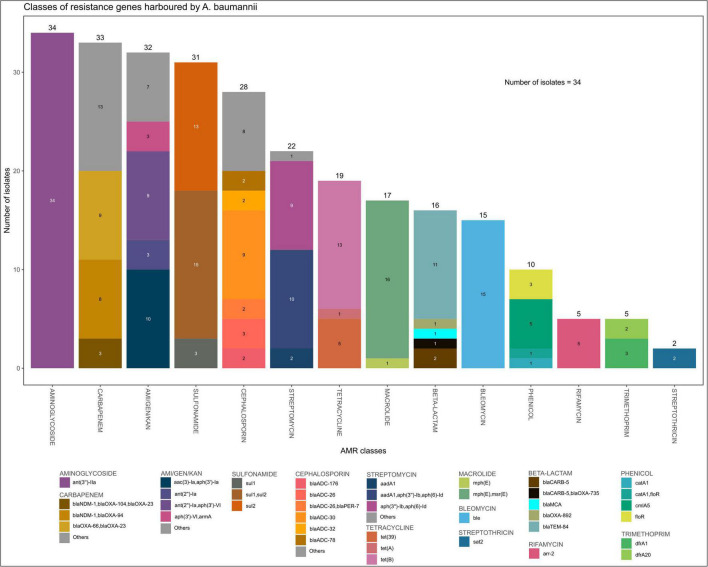
Resistance genes detected in *A. baumannii* isolates from OAUTCH, Ife, Osun State, and UCH, Ibadan, Oyo State, Nigeria, between 2017 and 2019. AMI, Amikacin; GEN, Gentamicin; KAN, Kanamycin.

**FIGURE 4:**
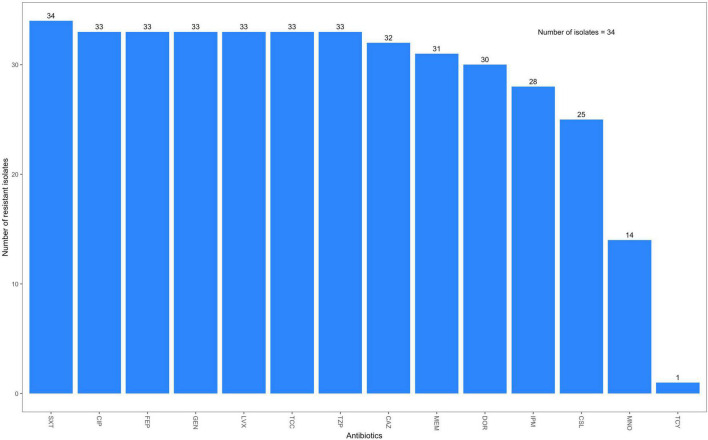
Phenotypic resistance of *A. baumannii* isolates from OAUTCH, Ife, Osun State, and UCH, Ibadan, Oyo State, Nigeria, between 2017 and 2019. SXT, trimethoprim/sulfamethoxazole; CIP, ciprofloxacin; FEP, cefepime; GEN, gentamicin; LVX, levofloxacin; TCC, ticarcillin/clavulanic acid; TZP, piperacillin/tazobactam; CAZ, ceftazidime; MEM, meropenem; DOR, doripenem; IPM, imipenem; CSL, cefoperazone/sulbactam; MNO, minocycline; TCY, tigecycline.

All 34 *A. baumannii* isolates harbored variants of the intrinsically encoded *blaOXA-51*-family carbapenemase gene (Figure 3 and Supplementary Table 2). In addition to these intrinsic *blaOXA*-*51* family genes, all but one of the *A. baumannii* isolates carried other carbapenem resistance genes. Seventeen (50.0%) isolates, including all IC1, IC2 (clade 1) and IC7 strains harbored variants of the potent *blaOXA-23*-like carbapenemase gene; four of these strains also harbored the *blaNDM-1* gene. *blaNDM-1* was present in 15 (44.1%) strains, including all eight ST1089 (clade 2) strains and two of the novel ST strains (ST2450 and ST2456). This ST2450 strain was highly resistant and was the only strain that carried the *blaOXA-58* carbapenemase gene. Two other strains carried the *blaOXA*-420 variant of the *blaOXA*-58 family. Phenotypically, thirty-one (91.2%) strains were resistant to at least one of the carbapenem antibiotics tested; 31 strains were resistant to meropenem, 30 were resistant to doripenem, and 28 were resistant to imipenem (Figure 4 and Supplementary Table 3).

Other beta-lactam resistance genes were found in most of the isolates. Several variants of the AmpC gene, *blaADC*, which is an intrinsic chromosomally encoded gene that confers cephalosporin resistance, were detected in 28 (82.4%) of the isolates. The remaining six isolates carried copies of the gene that were flagged by the ariba prediction software as being either “fragmented” or “interrupted,” indicating either a non-coding variant or a gene structure significantly different from that of the gene in the NCBI AMR database, respectively. Furthermore, all ten IC2 isolates, as well as the ST441 strain, harbored the broad-spectrum beta-lactamase gene, *blaTEM-84*. All *Acinetobacter* isolates harbored genes conferring streptomycin resistance. Amikacin, gentamicin and kanamycin resistance genes were also detected in 32 (94.1%) of the *A. baumannii* isolates. Thirty-one (91.2%) *A. baumannii* isolates also harbored at least one sulfonamide resistance gene; 15 isolates carried both *sul1 and sul2*. At least one of the tetracycline efflux transporter genes, *tetA*, *tetB*, and *tet39* was detected in 19 (55.9%) of the *A. baumannii* isolates. The resistance rate among *A. baumannii* strains in this study was very high, with >88% of the strains being resistant or intermediate to 10 of the 14 antibiotics tested, including the carbapenems.

The outbreak strains in clade 1 all harbored multiple resistance genes. All of them harbored the *blaOXA-23* gene and were all phenotypically resistant to doripenem, imipenem, and meropenem. They also harbored genes mediating resistance to streptomycin, beta-lactams/cephalosporins, sulfonamides, tetracycline, and other aminoglycosides, including amikacin, gentamicin and kanamycin, and were only sensitive to tigecycline amongst all the antibiotics tested.

## Discussion

Carbapenem-resistant *A. baumannii* are critically important nosocomial pathogens with limited treatment options. In this study, we confirmed an outbreak of CRAB belonging to IC2 and carrying multiple resistance determinants. We also demonstrated the preponderance of relatively unknown and previously undescribed CRAB lineages in our study area in South-west Nigeria.

Nearly a tenth of the patients acquired CRAB infections while admitted in the ICU during the study period, and CRAB acquisition was associated with higher odds of a subsequent bacterial bloodstream infection. As no CRAB was detected in swab samples collected from the ICU environment during the study period, we hypothesize that CRAB transmission may have occurred *via* health care workers. It is noteworthy that before this study, *A. baumannii* detection in the ICU of this hospital was almost non-existent due to limited diagnostic capacity and the difficulty in identifying *A. baumannii* using the conventional microbiological techniques in use. This was also exemplified in the number of *A. baumannii* isolates received from the AMR surveillance sentinel hospitals across Nigeria; only 16 *Acinetobacter* isolates were submitted to the AMR reference laboratory by the sentinel surveillance sites as part of their retrospective (2017–2019) collection. One limitation of the study was our inability to assess the differences in clonality and/or antimicrobial resistance phenotype or genotype between the community-acquired and hospital-acquired strains as information needed to robustly categorize the isolates was not available to us.

Our phylogenetic analyses revealed an *A. baumannii* outbreak in the ICU ward of OAUTHC. Six of the outbreak strains identified were isolated within 2 weeks of each other from six different patients within the same ICU unit. These strains belong to the IC2 lineage previously thought to be endemic in Europe and the United States (25) but increasingly reported as causes of nosocomial infections globally (12, 26–30). Strains in this lineage are highly adapted to the hospital setting, can spread rapidly within hospitals, and are highly resistant to antimicrobials, including carbapenems (12, 31).

One interesting observation was that the phylogenetically identical outbreak strains resolved into two different Oxford STs; ST1114 and ST1841 due to the presence of two *ghdB* loci in each of the strains. The *gdhB* gene has been described to be prone to duplication in *A. baumannii*, and indeed a previous study found that several artifactual and unreal *A. baumannii* STs exist in the Oxford PubMLST database due primarily to typing based on a second *gdhB* locus (32). As the replacement of the *gdhB* locus in the Oxford MLST scheme may not be feasible, exploiting long read or paired-end short-read data to identify the two *gdhB* loci during genome-based MLST is recommended. Although core genome MLST and core genome or whole genome SNP-based phylogenetic analyses are sufficient to resolve *A. baumannii* population structures, the Oxford MLST scheme remains important in the description of *A. baumannii* lineages due to its high discriminatory power. Cleaning artifactual STs from the database should be possible as has been described (32), and resolving this simple problem will streamline and facilitate MLST-based surveillance where whole genome sequencing resources are unavailable.

The IC2 strains identified as part of the outbreak made up a quarter of the *A. baumannii* isolates characterized in this study. Nevertheless, this prevalence may have been an overrepresentation caused by the outbreak. The ST1089 (IC9) strains were the second most common lineage in this study. These strains have been isolated from clinical samples and hospital environments, particularly from studies in Northern Africa and in the Middle East, and are frequently reported to carry *blaNDM-1* and *blaOXA-94* carbapenem resistance genes (23, 33–39). An ST1089 strain carrying *blaNDM-1* was also isolated from raw milk in a dairy farm in Algeria (40). All eight ST1089 strains in our study carried the *blaNDM-1* gene, as well as the *blaOXA-94* variant of the *blaOXA-51*-family carbapenemase genes. Although five of these strains were isolated in the ICU, these five strains were isolated over a 42-week period and shared between 6 and 207 SNP differences between them. The remaining three strains were isolated from OAUTHC, Ife, and UCH, Ibadan, and all eight strains differed by up to 213 SNPs, indicating that this clone may be endemic and circulating in Nigeria. About a third of the *A. baumannii* lineages in the hospital setting in this study were novel. A retrospective study that examined the *A. baumannii* lineages circulating and causing infections in Chile between 1990 and 2015 had similar findings, demonstrating that endemic clones different from those that had been described globally were predominant in Chilean settings but that these lineages were similar to those described elsewhere in South America (11). Our retrospective dataset is small, unrepresentative and insufficient to describe the epidemiology of *A. baumannii* lineages in Nigeria, but the available data does suggest that previously undescribed *A. baumannii* lineages may predominate in our setting.

*In silico* prediction of AMR determinants revealed most of the strains to be highly resistant, possessing resistance determinants to multiple antimicrobial classes. There was no difference in the number of resistance genes harbored between the outbreak strains and the other *A. baumannii* strains. Expectedly, all the *A. baumannii* isolates harbored variants of the intrinsic chromosomally encoded *blaOXA-51*-like gene, some of which may confer carbapenem resistance under certain conditions such as overexpression caused by the presence of an ISAba1 upstream of the gene (41). Carbapenem resistance in *A. baumannii* is most often mediated by acquired carbapenemase genes, particularly of the oxacillinase type, with *blaOXA*-*23*-like, *blaOXA*-*24*-like, *blaOXA*-*58*-like, *blaOXA*-*143*-like, and *blaOXA*-*235*-like genes being the most notable (2). Only *blaOXA*-*58*-like genes and *blaOXA*-*23*, which is the most widespread of the carbapenemase genes reported in clinical *A. baumannii* globally (42–46), were detected in the isolates in this study. *blaOXA*-58 had not previously been reported in Nigeria, and *blaOXA*-23 has been reported in only two Nigerian studies (15, 16). The *blaOXA-420* variant of the *blaOXA-58* family detected in one isolate has been reported in *A. baumannii* in only a handful of studies, including two conducted in neighboring Ghana (47–49). *blaNDM-1*, which is a less commonly described but highly potent carbapenem resistance gene in *A. baumannii*, was present in over a third of the isolates, four of which also co-carried *blaOXA*-23 (50). Carbapenem resistance in *A. baumannii* always translates to multidrug resistance, and often to extensive drug resistance (51), as evidenced by the high proportion of CRAB isolates carrying genes conferring resistance to other antibiotic classes. Thus, the high prevalence of mobilizable carbapenem resistance genes in this setting is worrying, particularly as the remaining treatment options (51) outside carbapenems are hugely limited in most hospitals in Nigeria as well as in other African countries. Furthermore, the true picture of *A. baumannii* prevalence and modes of spread in hospital settings in Nigeria remains unknown. As *A. baumannii* are known to spread primarily through clonal dissemination, we urgently need to identify contributors to their increasing spread in hospital settings in Nigeria. This would entail a holistic description of the endemic and circulating lineages, an identification of environmental reservoirs, if any, and a description of the infection prevention and control gaps that facilitate their spread in hospitals in our setting.

## Conclusion

We report the first description of IC2 *A. baumannii* strains causing an outbreak in an ICU in Nigeria. This study underscores the need to improve the capacity for the recovery and detection of *A. baumannii* in clinical samples in Nigeria and for intervention studies to mitigate CRAB transmission in Nigerian hospital settings.

## Data Availability Statement

The datasets presented in this study can be found in online repositories. The names of the repository/repositories and accession number(s) can be found below: https://www.ebi.ac.uk/ena, ERR4783259, ERR4783518, ERR4783200, ERR4 783180, ERR4783258, ERR4783419, ERR4783264, ERR4783252, ERR4783188, ERR4783391, ERR4783231, ERR4783186, ERR47 83194, ERR4783223, ERR4783203, ERR4783222, ERR4783387, ERR4783195, ERR4783401, ERR4783244, ERR4783250, ERR47 83214, ERR4783172, ERR4783228, ERR4783208, ERR6938084, ERR6938111, ERR6938087, ERR6938090, ERR6938114, ERR69 38093, ERR6938096, ERR6938099, ERR6938061, ERR478 3267, and ERR4783236.

## Ethics Statement

The studies involving human participants were reviewed and approved by Ethics and Research Committee of the Obafemi Awolowo University Teaching Hospitals Complex, Ife, Nigeria (protocol number: ERC/2017/06/13). The patients/participants provided their written informed consent to participate in this study.

## Author Contributions

EEO, EOI, INO, and AOAb conceptualized the study. EEO, EOI, AOO, AOAf, AU, SA, INO, and AOAb designed the analytical methods. EEO, EOI, TOO, AOO, ATA, and VOO contributed to the data collection and processing. EEO, EOI, and AOAf analyzed and interpreted the data. AOAf, AU, SA, AD, DMA, INO, and AOAb supervised the study. AD, DMA, INO, AOAb, EOI, TOO, ATA, and VOO provided resources or materials for the research. EEO drafted the manuscript. AD, AU, and INO critically reviewed the manuscript. All authors have read and approved the final version of the manuscript.

## Author Disclaimer

The views expressed in this publication are those of the authors and not necessarily those of the funders or their affiliates.

## Conflict of Interest

The authors declare that the research was conducted in the absence of any commercial or financial relationships that could be construed as a potential conflict of interest.

## Publisher’s Note

All claims expressed in this article are solely those of the authors and do not necessarily represent those of their affiliated organizations, or those of the publisher, the editors and the reviewers. Any product that may be evaluated in this article, or claim that may be made by its manufacturer, is not guaranteed or endorsed by the publisher.

## Footnotes

1
https://www.protocols.io/view/ghru-genomic-surveillance-of-antimicrobial-resista-bpn6mmhe

2
https://gitlab.com/antunderwood/bactinspector

3
https://gitlab.com/antunderwood/fastadist

4
http://www.phyloviz.net/goeburst/

5
https://www.ncbi.nlm.nih.gov/bioproject/PRJNA313047

6
http://www.who.int/drugresistance/whonetsoftware/en/

## Abbreviations

AMR: Antimicrobial resistance
CRAB: Carbapenem-resistant Acinetobacter baumannii
GHRU: Global Health Research Unit
IC: International clone
ICU: Intensive care unit
MLST: Multi-locus sequence typing
OAUTHC: Obafemi Awolowo University Teaching Hospitals Complex
OR: Odds ratio
SNP: Single nucleotide polymorphism
UCH: University College Hospital
VFDB: Virulence factor database
WGS: Whole genome sequencing.
