# Supplementary material for: Rectal Colonization and Nosocomial Transmission of Carbapenem-Resistant Acinetobacter baumannii in an Intensive Care Unit, Southwest Nigeria
Source: Front Med (Lausanne). 2022 Mar 7;9:846051. doi: 10.3389/fmed.2022.846051 (PMC8936076; doi:10.3389/fmed.2022.846051)
Supplement: Supplementary file 1 [file Data_Sheet_1.docx]

Supplementary Table 1: ST profiles of *Acinetobacter* isolates from OAUTHC, Ile-Ife, Osun State, Nigeria, UCH, Ibadan, Oyo State, Nigeria between 2017 and 2019.

| **ID** | **International clone (IC)** | **Oxford MLST** | | | | | | | | **Pasteur MLST** | | | | | | | |
| --- | --- | --- | --- | --- | --- | --- | --- | --- | --- | --- | --- | --- | --- | --- | --- | --- | --- |
|  |  | **gltA** | **gyrB** | **gdhB** | **recA** | **cpn60** | **gpi** | **rpoD** | **ST** | **cpn60** | **fusA** | **gltA** | **pyrG** | **recA** | **rplB** | **rpoB** | **ST** |
| G18584108 | - | 28 | 38 | 45 | 1 | 16 | 66 | 2 | 1089 | 5 | 2 | 4 | 1 | 3 | 3 | 4 | 85 |
| G18581040 | IC2 | 1 | 12 | 189 | 2 | 2 | 79 | 3 | 1841 | 2 | 2 | 2 | 2 | 2 | 2 | 2 | 2 |
| G18581047 | IC2 | 1 | 12 | 3 | 2 | 2 | 79 | 3 | 1114 | 2 | 2 | 2 | 2 | 2 | 2 | 2 | 2 |
| G18501059 | IC7 | 1 | 15 | 2 | 28 | 1 | 107 | 32 | 229 | 3 | 3 | 2 | 4 | 7 | 2 | 4 | 25 |
| G20500009 | - | 31 | 33 | 67 | 40 | 1 | 142 | 7 | 862 | 3 | 12 | 11 | 2 | 14 | 9 | 14 | 149 |
| G20500012 | - | 1 | 3 | 6 | 1 | 4 | 102 | 6 | 930 | 1 | 1 | 2 | 2 | 3 | 4 | 4 | 32 |
| G18581033 | IC2 | 1 | 12 | 3 | 2 | 2 | 79 | 3 | 1114 | 2 | 2 | 2 | 2 | 2 | 2 | 2 | 2 |
| G18581026 | IC2 | 1 | 12 | 189 | 2 | 2 | 79 | 3 | 1841 | 2 | 2 | 2 | 2 | 2 | 2 | 2 | 2 |
| G18581037 | IC2 | 1 | 12 | 189 | 2 | 2 | 79 | 3 | 1841 | 2 | 2 | 2 | 2 | 2 | 2 | 2 | 2 |
| G20500008 | - | 21 | 48 | 58 | 42 | 36 | 114 | 4 | 1418 | 40 | 3 | 7 | 2 | 40 | 4 | 4 | 164 |
| G18581010 | - | 1 | 64 | 109 | 1 | 23 | 153 | 26 | 2417 * | 12 | 37 | 2 | 2 | 3 | 2 | 14 | 267 |
| G20500013 | IC2 | 1 | 15 | 189 | 2 | 2 | 265 | 3 | 2456 * | 2 | 2 | 2 | 2 | 2 | 2 | 2 | 2 |
| G20500007 | IC1 | 10 | 12 | 182 | 2 | 4 | 79 | 5 | 2452 * | 1 | 1 | 1 | 1 | 2 | 1 | 1 | 409 |
| G18581066 | IC2 | 1 | 12 | 3 | 2 | 2 | 79 | 3 | 1114 | 2 | 2 | 2 | 2 | 2 | 2 | 2 | 2 |
| G18581045 | IC2 | 1 | 12 | 3 | 2 | 2 | 79 | 3 | 1114 | 2 | 2 | 2 | 2 | 2 | 2 | 2 | 2 |
| G18584110 | - | 28 | 38 | 45 | 1 | 16 | 66 | 2 | 1089 | 5 | 2 | 4 | 1 | 3 | 3 | 4 | 85 |
| G18581067 | - | 28 | 38 | 45 | 1 | 16 | 66 | 2 | 1089 | 5 | 2 | 4 | 1 | 3 | 3 | 4 | 85 |
| G20500011 | - | 33 | 12 | 212 | 31 | 32 | 188 | 45 | 2451 * | 8 | 1 | 5 | 26 | 42 | 2 | 3 | 1866 * |
| G20500010 | - | 31 | 33 | 67 | 40 | 1 | 142 | 7 | 862 | 3 | 12 | 11 | 2 | 14 | 9 | 14 | 149 |
| G18581042 | - | 1 | 107 | 166 | 6 | 23 | 285 | 26 | 2146 * | 12 | 100 | 2 | 2 | 9 | 1 | 52 | 821 |
| G18581070 | IC1 | 10 | 12 | 4 | 11 | 4 | 98 | 5 | 231 | 1 | 1 | 1 | 1 | 5 | 1 | 1 | 1 |
| G18584098 | IC1 | 10 | 12 | 4 | 11 | 4 | 100 | 5 | 441 | 1 | 1 | 1 | 1 | 5 | 1 | 1 | 1 |
| G18581021 | - | 28 | 38 | 45 | 1 | 16 | 66 | 2 | 1089 | 5 | 2 | 4 | 1 | 3 | 3 | 4 | 85 |
| G18581032 | - | 21 | 48 | 58 | 42 | 36 | 107 | 4 | 1957 | 40 | 3 | 7 | 2 | 40 | 4 | 4 | 164 |
| G18581038 | IC2 | 1 | 12 | 3 | 2 | 2 | 79 | 3 | 1114 | 2 | 2 | 2 | 2 | 2 | 2 | 2 | 2 |
| G18584099 | - | 1 | 15 | 12 | 134 | 4 | 163 | 4 | 2450 * | 1 | 158 | 2 | 2 | 165 | 1 | 2 | 1093 |
| G18581065 | - | 28 | 38 | 45 | 1 | 16 | 66 | 2 | 1089 | 5 | 2 | 4 | 1 | 3 | 3 | 4 | 85 |
| G18581049 | - | 28 | 38 | 45 | 1 | 16 | 66 | 2 | 1089 | 5 | 2 | 4 | 1 | 3 | 3 | 4 | 85 |
| G18581071 | IC7 | 1 | 15 | 2 | 28 | 1 | 107 | 32 | 229 | 3 | 3 | 2 | 4 | 7 | 2 | 4 | 25 |
| G18581034 | IC2 | 1 | 12 | 189 | 2 | 2 | 79 | 3 | 1841 | 2 | 2 | 2 | 2 | 2 | 2 | 2 | 2 |
| G20500006 | - | 1 | 15 | 11 | 48 | 18 | 114 | 43 | 2453 * | 7 | 7 | 2 | 2 | 8 | 4 | 4 | 16 |
| G18581027 | - | 28 | 38 | 45 | 1 | 16 | 66 | 2 | 1089 | 5 | 2 | 4 | 1 | 3 | 3 | 4 | 85 |
| G20500183 | - | 31 | 33 | 67 | 40 | 1 | 142 | 7 | 862 | 3 | 12 | 11 | 2 | 14 | 9 | 14 | 149 |
| G18503416 | - | 28 | 38 | 45 | 1 | 16 | 66 | 2 | 1089 | 5 | 2 | 4 | 1 | 3 | 3 | 4 | 85 |

Asterisks show novel newly assigned STs

Supplementary Table 2: Antimicrobial resistance gene alleles detected in *Acinetobacter* isolates from OAUTHC, Ile-Ife, Osun State, Nigeria, UCH, Ibadan, Oyo State, Nigeria between 2017 and 2019.

| **STRAIN ID** | ***aac1*** | ***aac3I*** | ***aac3II*** | ***aadA*** | ***aadA1+*** | ***ant2Ia*** | ***ant3IIa*** | ***aph3Ia*** | ***aph3VI*** | ***aph3Ib*** | ***aph6Id*** | ***armA*** | ***arr*** | ***blaADC*** | ***blaCARB*** | ***blaMCA*** | ***blaNDM*** | ***blaOXA21*** | ***blaOXA25*** | ***blaOXA4*** | ***blaPER*** | ***blaTEM*** | ***ble2*** | ***catA1*** | ***cmlA*** | ***dfrA1*** | ***dfrA20*** | ***ermB*** | ***floR*** | ***mphE*** | ***msrE*** | ***qacE*** | ***sat2+*** | ***str*** | ***sul1*** | ***sul2*** | ***tet39*** | ***tetA*** | ***tetB*** |
| --- | --- | --- | --- | --- | --- | --- | --- | --- | --- | --- | --- | --- | --- | --- | --- | --- | --- | --- | --- | --- | --- | --- | --- | --- | --- | --- | --- | --- | --- | --- | --- | --- | --- | --- | --- | --- | --- | --- | --- |
| **G18501059** | *aac6Ian* |  | *aac3IIe* |  |  |  | *ant3IIa* |  |  | *aph3Ib* | *aph6Id* | *armA* | *arr2* | *blaADC26* |  |  |  | *blaOXA64* | *blaOXA23* |  | *blaPER7* |  |  |  | *cmlA5* |  |  |  |  | *mphE* | *msrE* | *qacE* |  |  | *sul1* | *sul2* |  |  | *tetB* |
| **G18503416** |  |  |  |  |  | *ant2Ia* | *ant3IIa* |  | *aph3VI* |  |  |  |  | *blaADC80* |  |  | *blaNDM1* | *blaOXA94* |  |  |  |  | *ble* |  |  |  |  |  |  | *mphE* | *msrE* |  |  |  |  | *sul2* |  |  |  |
| **G18581010** |  |  |  |  |  |  | *ant3IIa* |  |  |  |  |  |  | *blaADC57* |  |  |  | *blaOXA180* |  |  |  |  |  |  |  |  |  |  |  |  |  |  |  |  |  |  |  |  |  |
| **G18581021** |  |  |  |  |  | *ant2Ia* | *ant3IIa* |  | *aph3VI* |  |  |  |  | *blaADC176* |  |  | *blaNDM1* | *blaOXA94* |  |  |  |  | *ble* |  |  |  |  |  |  | *mphE* | *msrE* |  |  |  |  | *sul2* |  |  |  |
| **G18581026** |  | *aac3Ia* |  | *aadA13* | *aadA1* |  | *ant3IIa* | *aph3Ia* |  | *aph3Ib* | *aph6Id* |  |  | *blaADC30* |  |  |  | *blaOXA66* | *blaOXA23* |  |  | *blaTEM84* |  |  |  |  |  |  |  |  |  | *qacEdelta1* |  |  | *sul1* | *sul2* |  |  | *tetB* |
| **G18581027** |  |  |  |  |  | *ant2Ia* | *ant3IIa* |  | *aph3VI* |  |  |  |  | *blaADC176* |  |  | *blaNDM1* | *blaOXA94* |  |  |  |  | *ble* |  |  |  |  |  | *floR* | *mphE* | *msrE* |  |  |  |  | *sul2* |  |  |  |
| **G18581032** |  |  |  |  |  | *ant2Ia* | *ant3IIa* |  |  |  |  |  |  | *blaADC199* | *blaCARB5* |  |  | *blaOXA91* |  |  |  |  |  |  |  |  |  |  |  |  |  |  |  |  |  |  | *tet39* |  |  |
| **G18581033** |  | *aac3Ia* |  | *aadA13* | *aadA1* |  | *ant3IIa* | *aph3Ia* |  | *aph3Ib* | *aph6Id* |  |  | *blaADC30* |  |  |  | *blaOXA66* | *blaOXA23* |  |  | *blaTEM84* |  |  |  |  |  |  |  |  |  | *qacEdelta1* |  |  | *sul1* | *sul2* |  |  | *tetB* |
| **G18581034** |  | *aac3Ia* |  | *aadA13* | *aadA1* |  | *ant3IIa* | *aph3Ia* |  | *aph3Ib* | *aph6Id* |  |  | *blaADC30* |  |  |  | *blaOXA66* | *blaOXA23* |  |  | *blaTEM84* |  |  |  |  |  |  |  |  |  | *qacEdelta1* |  |  | *sul1* | *sul2* |  |  | *tetB* |
| **G18581037** |  | *aac3Ia* |  | *aadA13* | *aadA1* |  | *ant3IIa* | *aph3Ia* |  | *aph3Ib* | *aph6Id* |  |  | *blaADC30* |  |  |  | *blaOXA66* | *blaOXA23* |  |  | *blaTEM84* |  |  |  |  |  |  |  |  |  | *qacEdelta1* |  |  | *sul1* | *sul2* |  |  | *tetB* |
| **G18581038** |  | *aac3Ia* |  | *aadA13* | *aadA1* |  | *ant3IIa* | *aph3Ia* |  | *aph3Ib* | *aph6Id* |  |  | *blaADC30* |  |  |  | *blaOXA66* | *blaOXA23* |  |  | *blaTEM84* |  |  |  |  |  |  |  |  |  | *qacEdelta1* |  |  | *sul1* | *sul2* |  |  | *tetB* |
| **G18581040** |  | *aac3Ia* |  | *aadA13* | *aadA1* |  | *ant3IIa* | *aph3Ia* |  | *aph3Ib* | *aph6Id* |  |  | *blaADC30* |  |  |  | *blaOXA66* | *blaOXA23* |  |  | *blaTEM84* |  |  |  |  |  |  |  |  |  | *qacEdelta1* |  |  | *sul1* | *sul2* |  |  | *tetB* |
| **G18581042** |  |  |  |  |  | *ant2Ia* | *ant3IIa* | *aph3Ia* |  | *aph3Ib* | *aph6Id* |  |  | *blaADC32* |  | *blaMCA* |  | *blaOXA378* |  | *blaOXA420* |  |  |  |  |  |  | *dfrA20* |  |  | *mphE* | *msrE* |  |  |  |  | *sul2* | *tet39* |  |  |
| **G18581045** |  | *aac3Ia* |  | *aadA13* | *aadA1* |  | *ant3IIa* | *aph3Ia* |  | *aph3Ib* | *aph6Id* |  |  | *blaADC30* |  |  |  | *blaOXA66* | *blaOXA23* |  |  | *blaTEM84* |  |  |  |  |  |  |  |  |  | *qacEdelta1* |  |  | *sul1* | *sul2* |  |  | *tetB* |
| **G18581047** |  | *aac3Ia* |  | *aadA13* | *aadA1* |  | *ant3IIa* | *aph3Ia* |  | *aph3Ib* | *aph6Id* |  |  | *blaADC30* |  |  |  | *blaOXA66* | *blaOXA23* |  |  | *blaTEM84* |  |  |  |  |  |  |  |  |  | *qacEdelta1* |  |  | *sul1* | *sul2* |  |  | *tetB* |
| **G18581049** |  |  |  |  |  | *ant2Ia* | *ant3IIa* |  | *aph3VI* |  |  |  |  | *blaADC176* |  |  | *blaNDM1* | *blaOXA94* |  |  |  |  | *ble* |  |  |  |  |  | *floR* | *mphE* | *msrE* |  |  |  |  | *sul2* |  |  |  |
| **G18581065** |  |  |  |  |  | *ant2Ia* | *ant3IIa* |  | *aph3VI* |  |  |  |  | *blaADC176* |  |  | *blaNDM1* | *blaOXA94* |  |  |  |  | *ble* |  |  |  |  |  |  | *mphE* | *msrE* |  |  |  |  | *sul2* |  |  |  |
| **G18581066** |  | *aac3Ia* |  | *aadA13* | *aadA1* |  | *ant3IIa* | *aph3Ia* |  | *aph3Ib* | *aph6Id* |  |  | *blaADC30* |  |  |  | *blaOXA66* | *blaOXA23* |  |  | *blaTEM84* |  |  |  |  |  |  |  |  |  | *qacEdelta1* |  |  | *sul1* | *sul2* |  |  | *tetB* |
| **G18581067** |  |  |  |  |  | *ant2Ia* | *ant3IIa* | *aph3Ia* | *aph3VI* |  |  |  |  | *blaADC234* |  |  | *blaNDM1* | *blaOXA94* |  |  |  |  | *ble* |  |  |  |  |  |  | *mphE* | *msrE* |  |  |  |  | *sul2* |  |  |  |
| **G18581070** |  | *aac3Ia* |  | *aadA13* | *aadA1* |  | *ant3IIa* | *aph3Ia* | *aph3VIa* | *aph3Ib* | *aph6Id* |  |  | *blaADC191* |  |  | *blaNDM1* | *blaOXA69* | *blaOXA23* |  |  |  | *ble* |  |  | *dfrA1* |  |  |  |  |  | *qacEdelta1* | *sat2* |  | *sul1* | *sul2* |  |  | *tetB* |
| **G18581071** | *aac6Ian* |  | *aac3IIe* |  |  |  | *ant3IIa* |  |  | *aph3Ib* | *aph6Id* | *armA* | *arr2* | *blaADC26* |  |  |  | *blaOXA64* | *blaOXA23* |  | *blaPER7* |  |  |  | *cmlA5* |  |  |  |  | *mphE* | *msrE* | *qacE* |  |  | *sul1* | *sul2* |  |  | *tetB* |
| **G18584098** |  | *aac3Ia* |  | *aadA13* | *aadA1* |  | *ant3IIa* | *aph3Ia* |  |  |  |  |  | *blaADC78* |  |  |  | *blaOXA69* | *blaOXA23* |  |  | *blaTEM84* |  | *catA1* |  |  |  |  |  |  |  | *qacEdelta1* |  |  | *sul1* |  |  | *tetA* |  |
| **G18584099** |  |  |  |  |  | *ant2Ia* | *ant3IIa* |  |  | *aph3Ib* | *aph6Id* |  |  | *blaADC163* | *blaCARB5* |  | *blaNDM1* | *blaOXA735* |  | *blaOXA58* |  |  | *ble* |  |  | *dfrA1* |  |  | *floR* | *mphE* | *msrE* |  |  |  |  | *sul2* | *tet39* |  |  |
| **G18584108** |  |  |  |  |  | *ant2Ia* | *ant3IIa* |  | *aph3VI* |  |  |  |  | *blaADC176* |  |  | *blaNDM1* | *blaOXA94* |  |  |  |  | *ble* |  |  |  |  |  | *floR* | *mphE* | *msrE* |  |  |  |  | *sul2* |  |  |  |
| **G18584110** |  |  |  |  |  | *ant2Ia* | *ant3IIa* |  | *aph3VI* |  |  |  |  | *blaADC199* |  |  | *blaNDM1* | *blaOXA94* |  |  |  |  | *ble* |  |  |  |  |  |  | *mphE* | *msrE* |  |  |  |  | *sul2* |  |  |  |
| **G20500006** |  |  |  |  |  | *ant2Ia* | *ant3IIa* | *aph3Ia* |  | *aph3Ib* | *aph6Id* |  |  | *blaADC169* |  |  |  | *blaOXA402* |  | *blaOXA420* |  |  |  |  |  |  | *dfrA20* |  |  | *mphE* | *msrE* |  |  |  |  | *sul2* | *tet39* |  |  |
| **G20500007** |  | *aac3Ia* |  | *aadA13* | *aadA1* |  | *ant3IIa* |  | *aph3VIa* |  |  |  |  | *blaADC78* |  |  |  | *blaOXA69* | *blaOXA23* |  |  |  |  |  |  |  |  |  |  |  |  | *qacEdelta1* |  |  | *sul1* |  |  |  |  |
| **G20500008** |  |  |  |  |  | *ant2Ia* | *ant3IIa* |  |  |  |  |  |  | *blaADC199* | *blaCARB5* |  |  | *blaOXA91* |  |  |  |  |  |  |  |  |  |  |  |  |  |  |  |  |  | *sul2* | *tet39* |  |  |
| **G20500009** |  |  |  |  |  |  | *ant3IIa* |  | *aph3VI* | *aph3Ib* | *aph6Id* | *armA* | *arr2* | *blaADC26* |  |  | *blaNDM1* | *blaOXA104* | *blaOXA23* |  |  |  | *ble* |  | *cmlA5* |  |  |  |  | *mphE* | *msrE* | *qacEdelta1* |  |  | *sul1* | *sul2* |  |  |  |
| **G20500010** |  |  |  |  |  |  | *ant3IIa* |  | *aph3VI* | *aph3Ib* | *aph6Id* | *armA* | *arr2* | *blaADC26* |  |  | *blaNDM1* | *blaOXA104* | *blaOXA23* |  |  |  | *ble* |  | *cmlA5* |  |  |  |  | *mphE* | *msrE* | *qacEdelta1* |  |  | *sul1* | *sul2* |  |  |  |
| **G20500011** |  |  |  |  |  |  | *ant3IIa* |  |  |  |  |  |  | *blaADC32* |  |  |  | *blaOXA892* |  |  |  |  |  |  |  |  |  |  |  |  |  |  |  |  |  |  |  |  |  |
| **G20500012** |  |  |  |  |  | *ant2Ia* | *ant3IIa* |  | *aph3VI* | *aph3Ib* | *aph6Id* |  |  | *blaADC79* |  |  | *blaNDM1* | *blaOXA100* |  |  |  |  | *ble* |  |  |  |  |  |  |  |  |  |  |  |  | *sul2* |  |  |  |
| **G20500013** |  | *aac3Ia* | *aac3IId* | *aadA13* | *aadA1* |  | *ant3IIa* | *aph3Ia* |  | *aph3Ib* | *aph6Id* |  |  | *blaADC25* |  |  | *blaNDM1* | *blaOXA66* |  |  |  | *blaTEM84* | *ble* | *catA1* |  | *dfrA1* |  | *ermB* | *floR* | *mphE* | *msrE* | *qacEdelta1* | *sat2* | *str* | *sul1* |  |  |  | *tetB* |
| **G20500183** |  |  |  |  |  |  | *ant3IIa* |  | *aph3VI* | *aph3Ib* | *aph6Id* | *armA* | *arr2* | *blaADC26* |  |  | *blaNDM1* | *blaOXA104* | *blaOXA23* |  |  |  | *ble* |  | *cmlA5* |  |  |  |  | *mphE* | *msrE* | *qacEdelta1* |  |  | *sul1* | *sul2* |  |  |  |

Supplementary Table 3: Phenotypic antimicrobial resistance profiles of *Acinetobacter* isolates from OAUTHC, Ile-Ife, Osun State, Nigeria, UCH, Ibadan, Oyo State, Nigeria between 2017 and 2019.

| id | TCC | TZP | CAZ | CSL | FEP | DOR | IPM | MEM | GEN | CIP | LVX | MNO | TCY | SXT |
| --- | --- | --- | --- | --- | --- | --- | --- | --- | --- | --- | --- | --- | --- | --- |
| G18501059 | >=128 (R) | >=128 (R) | >=64 (R) | 32 (I) | >=64 (R) | >=8 (R) | >=16 (R) | >=16 (R) | >=16 (R) | >=4 (R) | 4 (R) | <=1 (S) | <=0.5 (S) | >=320 (R) |
| G18503416 | >=128 (R) | >=128 (R) | >=64 (R) | 32 (I) | >=64 (R) | >=8 (R) | >=16 (R) | 8 (R) | 8 (I) | >=4 (R) | >=8 (R) | <=1 (S) | <=0.5 (S) | >=320 (R) |
| G18581010 | >=128 (R) | >=128 (R) | >=64 (R) | >=64 (R) | 32 (R) | >=8 (R) | >=16 (R) | >=16 (R) | >=16 (R) | >=4 (R) | >=8 (R) | 4 (S) | 2 (S) | >=320 (R) |
| G18581021 | >=128 (R) | >=128 (R) | >=64 (R) | >=64 (R) | >=64 (R) | >=8 (R) | >=16 (R) | >=16 (R) | 8 (I) | >=4 (R) | >=8 (R) | 8 (I) | 4 (S) | >=320 (R) |
| G18581026 | >=128 (R) | >=128 (R) | >=64 (R) | >=64 (R) | >=64 (R) | >=8 (R) | >=16 (R) | >=16 (R) | 8 (I) | >=4 (R) | >=8 (R) | 8 (I) | 4 (S) | >=320 (R) |
| G18581027 | >=128 (R) | >=128 (R) | >=64 (R) | 16 (S) | >=64 (R) | 0.25 (S) | <=0.25 (S) | 8 (R) | >=16 (R) | >=4 (R) | >=8 (R) | 8 (I) | 2 (S) | >=320 (R) |
| G18581032 | >=128 (R) | 64 (I) | >=64 (R) | <=8 (S) | >=64 (R) | 0.5 (S) | 0.5 (S) | 1 (S) | >=16 (R) | >=4 (R) | 4 (R) | <=1 (S) | 1 (S) | <=20 (R) |
| G18581033 | >=128 (R) | >=128 (R) | >=64 (R) | 32 (I) | 32 (R) | >=8 (R) | 8 (R) | >=16 (R) | >=16 (R) | >=4 (R) | >=8 (R) | 8 (I) | 2 (S) | >=320 (R) |
| G18581034 | >=128 (R) | >=128 (R) | >=64 (R) | >=64 (R) | >=64 (R) | >=8 (R) | >=16 (R) | >=16 (R) | 8 (I) | >=4 (R) | >=8 (R) | 8 (I) | 4 (S) | >=320 (R) |
| G18581037 | >=128 (R) | >=128 (R) | >=64 (R) | >=64 (R) | >=64 (R) | >=8 (R) | >=16 (R) | >=16 (R) | 8 (I) | >=4 (R) | >=8 (R) | 8 (I) | 4 (S) | >=320 (R) |
| G18581038 | >=128 (R) | >=128 (R) | >=64 (R) | >=64 (R) | >=64 (R) | >=8 (R) | >=16 (R) | >=16 (R) | 8 (I) | >=4 (R) | >=8 (R) | 8 (I) | 4 (S) | >=320 (R) |
| G18581040 | >=128 (R) | >=128 (R) | >=64 (R) | >=64 (R) | >=64 (R) | >=8 (R) | >=16 (R) | >=16 (R) | 8 (I) | >=4 (R) | >=8 (R) | 8 (I) | 4 (S) | 160 (R) |
| G18581042 | >=128 (R) | >=128 (R) | 16 (R) | <=8 (S) | 16 (R) | 2 (I) | 1 (S) | 4 (R) | >=16 (R) | >=4 (R) | >=8 (R) | <=1 (S) | 2 (S) | >=320 (R) |
| G18581045 | >=128 (R) | >=128 (R) | >=64 (R) | >=64 (R) | >=64 (R) | >=8 (R) | >=16 (R) | >=16 (R) | 8 (I) | >=4 (R) | >=8 (R) | 8 (I) | 4 (S) | >=320 (R) |
| G18581047 | >=128 (R) | >=128 (R) | >=64 (R) | >=64 (R) | >=64 (R) | >=8 (R) | >=16 (R) | >=16 (R) | 8 (I) | >=4 (R) | >=8 (R) | 8 (I) | 4 (S) | >=320 (R) |
| G18581049 | >=128 (R) | >=128 (R) | >=64 (R) | 32 (I) | >=64 (R) | >=8 (R) | >=16 (R) | >=16 (R) | 8 (I) | >=4 (R) | >=8 (R) | <=1 (S) | <=0.5 (S) | >=320 (R) |
| G18581065 | >=128 (R) | >=128 (R) | >=64 (R) | 16 (S) | >=64 (R) | >=8 (R) | 8 (R) | 8 (R) | 8 (I) | >=4 (R) | >=8 (R) | <=1 (S) | <=0.5 (S) | >=320 (R) |
| G18581066 | >=128 (R) | >=128 (R) | >=64 (R) | >=64 (R) | >=64 (R) | >=8 (R) | >=16 (R) | >=16 (R) | 8 (I) | >=4 (R) | >=8 (R) | 8 (I) | 4 (S) | >=320 (R) |
| G18581067 | >=128 (R) | >=128 (R) | >=64 (R) | 32 (I) | >=64 (R) | >=8 (R) | 8 (R) | >=16 (R) | >=16 (R) | >=4 (R) | >=8 (R) | <=1 (S) | 1 (S) | >=320 (R) |
| G18581070 | >=128 (R) | >=128 (R) | >=64 (R) | >=64 (R) | >=64 (R) | >=8 (R) | >=16 (R) | >=16 (R) | 8 (I) | >=4 (R) | 4 (R) | 8 (I) | 4 (S) | >=320 (R) |
| G18581071 | >=128 (R) | >=128 (R) | >=64 (R) | 16 (S) | >=64 (R) | >=8 (R) | >=16 (R) | >=16 (R) | >=16 (R) | >=4 (R) | >=8 (R) | 2 (S) | 1 (S) | >=320 (R) |
| G18584098 | >=128 (R) | >=128 (R) | >=64 (R) | >=64 (R) | 32 (R) | >=8 (R) | >=16 (R) | >=16 (R) | >=16 (R) | >=4 (R) | >=8 (R) | >=16 (R) | 4 (S) | >=320 (R) |
| G18584099 | >=128 (R) | >=128 (R) | >=64 (R) | >=64 (R) | >=64 (R) | >=8 (R) | >=16 (R) | >=16 (R) | >=16 (R) | >=4 (R) | >=8 (R) | 8 (I) | 4 (S) | >=320 (R) |
| G18584108 | >=128 (R) | >=128 (R) | >=64 (R) | 32 (I) | >=64 (R) | >=8 (R) | 8 (R) | 8 (R) | >=16 (R) | >=4 (R) | >=8 (R) | <=1 (S) | 4 (S) | >=320 (R) |
| G18584110 | >=128 (R) | >=128 (R) | >=64 (R) | >=64 (R) | >=64 (R) | >=8 (R) | 8 (R) | >=16 (R) | >=16 (R) | >=4 (R) | >=8 (R) | <=1 (S) | 4 (S) | >=320 (R) |
| G20500006 | >=128 (R) | >=128 (R) | 4 (S) | <=8 (S) | 8 (I) | 2 (I) | 1 (S) | 2 (I) | >=16 (R) | >=4 (R) | >=8 (R) | 2 (S) | >=8 (R) | >=320 (R) |
| G20500007 | >=128 (R) | >=128 (R) | >=64 (R) | <=8 (S) | 32 (R) | >=8 (R) | 4 (R) | 8 (R) | >=16 (R) | >=4 (R) | 4 (R) | <=1 (S) | 2 (S) | >=320 (R) |
| G20500008 | >=128 (R) | 64 (I) | >=64 (R) | <=8 (S) | >=64 (R) | 0.5 (S) | 1 (S) | 0.5 (S) | >=16 (R) | >=4 (R) | 4 (R) | <=1 (S) | 1 (S) | 160 (R) |
| G20500009 | >=128 (R) | >=128 (R) | >=64 (R) | 32 (I) | >=64 (R) | >=8 (R) | >=16 (R) | >=16 (R) | >=16 (R) | >=4 (R) | 4 (R) | <=1 (S) | 1 (S) | >=320 (R) |
| G20500010 | >=128 (R) | >=128 (R) | >=64 (R) | >=64 (R) | >=64 (R) | >=8 (R) | >=16 (R) | >=16 (R) | >=16 (R) | >=4 (R) | >=8 (R) | <=1 (S) | 2 (S) | >=320 (R) |
| G20500011 | <=8 (S) | <=4 (S) | 4 (S) | <=8 (S) | 2 (S) | <=0.12 (S) | <=0.25 (S) | <=0.25 (S) | <=1 (S) | <=0.25 (S) | <=0.12 (S) | <=1 (S) | <=0.5 (S) | <=20 (R) |
| G20500012 | >=128 (R) | >=128 (R) | >=64 (R) | 32 (I) | >=64 (R) | >=8 (R) | 8 (R) | 8 (R) | >=16 (R) | >=4 (R) | 4 (R) | <=1 (S) | <=0.5 (S) | 160 (R) |
| G20500013 | >=128 (R) | >=128 (R) | >=64 (R) | >=64 (R) | >=64 (R) | >=8 (R) | 8 (R) | 8 (R) | >=16 (R) | >=4 (R) | >=8 (R) | 2 (S) | 1 (S) | >=320 (R) |
| G20500183 | >=128 (R) | >=128 (R) | >=64 (R) | 32 (I) | >=64 (R) | >=8 (R) | >=16 (R) | >=16 (R) | >=16 (R) | >=4 (R) | 4 (R) | <=1 (S) | 1 (S) | >=320 (R) |

SXT - trimethoprim/sulfamethoxazole; CIP – ciprofloxacin; FEP – cefepime; GEN – gentamicin; LVX – levofloxacin; TCC – ticarcillin/Clavulanic acid; TZP – piperacillin/tazobactam; CAZ – ceftazidime; MEM – meropenem; DOR – doripenem; IPM – imipenem; CSL – cefoperazone/sulbactam; MNO – minocycline; TCY – tigecycline.
